# A retrospective analysis to identify predictors of COPD-related rehospitalization

**DOI:** 10.1186/s12890-016-0231-3

**Published:** 2016-04-30

**Authors:** Melissa H. Roberts, Emmanuelle Clerisme-Beaty, Chris M. Kozma, Andrew Paris, Terra Slaton, Douglas W. Mapel

**Affiliations:** Lovelace Clinic Foundation, 2309 Renard Place SE, Suite 103, Albuquerque, NM 87106 USA; Boehringer Ingelheim Pharmaceuticals Inc., Ridgefield, CT USA; CK Consulting, St. Helena Island, SC USA; Vigilytics LLC, Victor, NY USA; TLS Statistics, West Columbia, SC USA

**Keywords:** Chronic obstructive pulmonary disease (COPD), Comorbidity, Exacerbations, Readmission predictors, Rehospitalization, Utilization

## Abstract

**Background:**

Chronic obstructive pulmonary disease (COPD) is often associated with recurrent hospitalizations. This study aimed to identify factors related to COPD rehospitalization.

**Methods:**

A national US claims database was used to identify patients, aged ≥40 years, hospitalized for COPD. Their first COPD-related hospital admission date in 2009 was set as the index date, with post-discharge COPD-related rehospitalization assessed for 180 days post-index date. Data were analyzed for: 1) all eligible patients in whom early COPD-related rehospitalization was evaluated (1–30 days post discharge; all-patient cohort) and 2) a patient subset not rehospitalized early in whom late COPD-related rehospitalization was evaluated (>30 days post discharge to 180 days post-index date; late cohort). Logistic regressions controlling for age and sex assessed potential COPD-related rehospitalization predictors. Variables from the 360-day pre-index period and index hospitalization were evaluated for each cohort, and 30-day post-discharge variables evaluated for the late cohort.

**Results:**

Of 3612 patients with an index hospitalization, 4.8 % (174) had an early COPD-related rehospitalization, and of the remaining 3438 patients, 13.7 % (471) had a late COPD-related rehospitalization. Several pre-index variables were predictive of early COPD-related rehospitalization including: pneumonia; comorbidities; COPD-related drug therapies; and prior hospitalizations. In patients not rehospitalized early, the strongest predictor of late COPD-related rehospitalization was pre-index COPD-related hospitalization (OR = 3.64 [*P* < 0.001]). The strongest index hospitalization factors predictive of late COPD-related rehospitalization were use of steroids (any route: OR = 1.62 [*P* = 0.007]) and nebulizers (OR = 1.65 [*P* = 0.007]); neither predicted early COPD-related rehospitalization. Generally, factors predicting COPD-related rehospitalization were similar in both cohorts.

**Conclusions:**

Several pre-index variables were associated with COPD-related rehospitalization. A strong predictor of COPD-related rehospitalization was prior hospitalization during the pre-index period, particularly with a primary COPD diagnosis, whilst other predictive factors related to increased COPD severity; these may be useful indicators for COPD-related rehospitalization risk assessment. Some factors, e.g., recurrent pneumonia and exacerbations, may be modifiable.

**Electronic supplementary material:**

The online version of this article (doi:10.1186/s12890-016-0231-3) contains supplementary material, which is available to authorized users.

## Background

Chronic obstructive pulmonary disease (COPD) is characterized by persistent airflow limitation and is associated with an enhanced chronic inflammatory response in the lungs to noxious particles or gases [1]. Although the primary effect of COPD is on the lungs, it is also associated with the development of adverse systemic effects and comorbidities, which contribute to its severity [2–4]. COPD is a major cause of chronic morbidity and mortality [1]; worldwide in 2010, it affected more than 210 million individuals [5] and was one of the top six leading causes of death [6]. In 2015, the World Health Organization estimated that approximately 65 million individuals had moderate-to-severe COPD and projected it to become the third leading cause of death globally by 2030 [7]. In 2012, chronic lower respiratory diseases, including COPD, were the third leading cause of deaths in the United States [8] and, in 2013, an estimated 15.7 million adults had physician-diagnosed COPD [9]. In 2012, the National Heart, Lung, and Blood Institute (NHLBI) estimated that around 12 million individuals in the United States had undiagnosed COPD [10].

Patients with COPD often experience acute worsening of respiratory symptoms, known as exacerbations, which range in severity from mild events that patients manage themselves at home to severe episodes that require hospitalization. Recurrent COPD exacerbations, particularly those requiring hospitalization, have been shown to hasten lung function decline and increase patient mortality, as well as have a deleterious impact on health-related quality of life [11–13]. According to the American Thoracic Society/European Respiratory Society task force [14], indications for hospitalization of patients with an exacerbation include: occurrence of high-risk comorbidities (e.g., pneumonia, cardiac arrhythmia, congestive heart failure, diabetes mellitus, renal or liver failure); inadequate response of symptoms to outpatient management; marked increase in dyspnea; inability to eat or sleep because of symptoms; worsening hypoxemia or hypercapnia; alterations in mental status; inability of patients to care for themselves (lack of home support); uncertain diagnosis; and inadequate home care.

COPD-related hospitalizations place a substantial economic burden on healthcare systems, and account for a large portion of the direct medical costs that are covered from a payer perspective [10]. In the United States, medical costs attributable to COPD were estimated at US $32.1 billion in 2010, and with current growth are predicted to rise to US $49.0 billion by 2020 [15]. COPD is also one of the major causes of early rehospitalization; approximately one in every five Medicare patients hospitalized for COPD have an all-cause rehospitalization within 30 days of discharge and around a third of these patients are readmitted for a COPD-related event [16]. Healthcare policy makers have identified reducing hospital readmissions as a way of improving healthcare quality while reducing costs, and in the United States the main public health insurance system has implemented a policy wherein hospitals will be penalized for rehospitalizations within 30 days after hospitalization for COPD [17]. This policy has been implemented despite a lack of specific interventions proven to reduce COPD-related rehospitalization [18]. Hospitals are now keenly interested in identifying patients with COPD who are at highest risk for hospital readmission, and in finding interventions that reduce rehospitalization risk.

The purpose of this study was to identify factors associated with COPD-related rehospitalization post discharge from an index hospitalization for COPD using variables that are typically available in administrative claims or encounter databases. We were particularly interested in the following characteristics: 1) demographic factors; 2) comorbid conditions in the pre-index period (360 days prior to the index admission); 3) use of COPD-related drugs in the pre-index period; 4) COPD-related and all-cause healthcare utilization in the pre-index period; 5) treatments during the index hospitalization; and 6) office visits and antibiotic or oral steroid treatments in the month after the index hospitalization. We stratified the post-index hospitalization period into two time periods: early (within 1–30 days post discharge) and late (>30 days post discharge to the end of the post-index period [i.e., 180 days from the index admission]), which allows examination of factors most relevant to those dealing with recent mandates while also allowing comparison with previous analyses. We chose to focus solely on COPD-related rehospitalizations, rather than all-cause rehospitalizations, in order to identify potentially modifiable COPD-related risk factors.

## Methods

### Data source

Data for this analysis were from SDI Health, which was acquired by IMS Health in 2011. The sources of SDI data are from national US claims clearinghouses and facilities including both hospital inpatient and outpatient details. According to SDI, at the time of data extraction there were nearly 20 million discharges captured annually with 4.5 million of these also capturing physician office-based encounters and nearly 4 million with office and prescription coverage (personal communication). All available hospital, office visit, and outpatient pharmacy claims were obtained for patients meeting the analysis criteria.

The retrospective SDI patient claims data were de-identified to ensure compliance with the Health Insurance Portability and Accountability Act (HIPAA) Privacy Rule standard for de-identification. Therefore, neither institutional review board approval nor informed patient consent were required for this study.

### Observation periods

The initial data extraction selected patients with any COPD-related diagnosis in 2008 through 2011. The time period of January 1, 2009 through December 31, 2009 was used to identify patients who had an inpatient hospitalization for COPD (i.e., the index event). The date of hospital admission was set as the index date and the post-index period was the first 180 days from the index admission. The SDI data did not include any information regarding health insurance coverage eligibility, which is frequently used in administrative database analyses to ensure completeness of the data. Therefore, proxy eligibility was required, which was established by claim activity (one pharmacy and one medical) for any reason between −720 and −361 days relative to the index date (i.e., pre-period eligibility indicator) and between +181 to +720 days relative to the index date (i.e., post-period eligibility indicator).

**Fig. 1:**
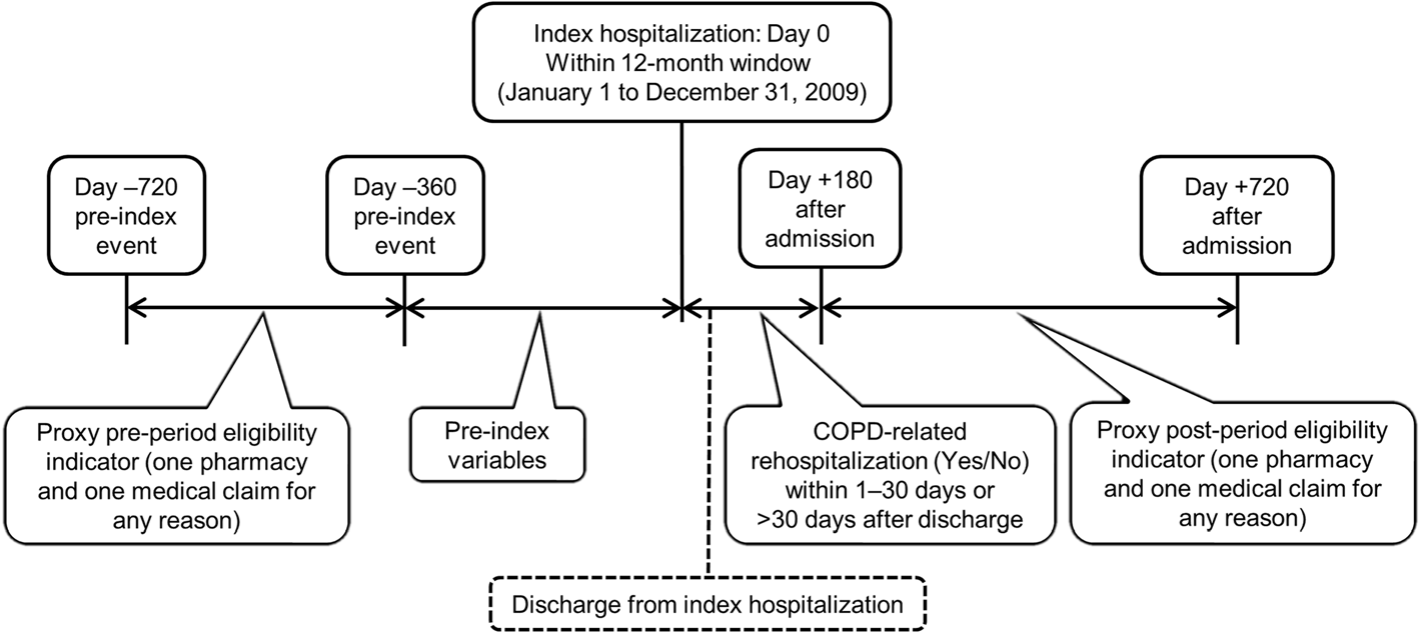
Study timeline and design

Two time periods for evaluating COPD-related rehospitalization were based on the discharge date from the index hospitalization; 1–30 days post discharge and >30 days post discharge to the end of the post-index period. A 360-day pre-index period and the index hospital event were used for identifying pre-index and in-hospital predictors. The first 30 days following discharge from the index hospitalization was also used for identifying predictors for patients whose first COPD-related rehospitalization occurred later in the observation period. A diagram showing the analysis observation periods is presented in Fig. 1.

### Analysis population

Patients were included in the study if they had a COPD-related hospitalization within the enrolment window (index event), were aged ≥40 years at the time of the index event, and met the proxy eligibility requirement. It was assumed that patients who met the proxy eligibility criterion had complete medical, pharmacy, and hospital data for the analysis period (−360 days before and 180 days after index hospitalization). COPD-related hospitalization was defined based on International Classification of Diseases, 9th Revision, Clinical Modification (ICD-9-CM) codes (491.xx–Chronic bronchitis; 492.xx–Emphysema; 493.2x–Chronic obstructive asthma, or 496-Chronic airway obstruction, not elsewhere classified).

Patients were excluded if they had: incomplete or invalid data for variables used in the analysis; a documented diagnosis of pregnancy, malignant neoplasm, or cystic fibrosis; hospice claims; an indication of multiple hospitalizations on the index date; index hospitalization costs ≥ $270,000; or lung transplant claims prior to the index date. The goal of the exclusion criteria was to remove patients who had non-COPD conditions that would influence hospitalizations ultimately affecting the rehospitalization rate.

### Primary endpoint

The primary endpoint for the study was the first COPD-related rehospitalization post discharge from the index event. In order to place this endpoint in context, the percentages of patients rehospitalized for any reason within 30 days of discharge and within 180 days of discharge were also calculated.

### Data analysis

#### Analysis groups

Analyses were conducted on two data sets. The first data set, referred to as the ‘all-patient cohort’, consisted of all eligible study patients, and was used to investigate the predictors of early COPD-related rehospitalization (i.e., within 1–30 days post discharge). The second data set, referred to as the ‘late cohort’, consisted of all eligible study patients except those who had their first COPD-related rehospitalization within 30 days post discharge. This second data set was used to investigate the predictors of late COPD-related rehospitalization (i.e., from >30 days post discharge to the end of the post-index period).

#### Coding

Operational definitions were developed for study variables from the 360-day pre-index period and for the in-hospital data. The in-hospital data consisted of Charge Description Master entries, which detail items billed to patients in an inpatient setting. Charge Description Master entries were qualitatively reviewed, and entries potentially related to COPD were coded. For the in-hospital data, the following types of variables were coded indicating presence or use: comorbidities; vaccines; smoking or history of smoking; transplantation; oxygen use; antibiotics; inhaled and oral steroids; xanthines; and oral and inhaled bronchodilators. For healthcare utilization other than inpatient, study variables were assessed using codes for ICD-9-CM, Current Procedural Terminology (CPT), Healthcare Common Procedure Coding System (HCPCS), and drug categories based on National Drug Codes (NDC). These variables included patients’ demographic data, comorbidities determined either by diagnosis or prescriptions, pre-index medications (long-acting beta-agonists [LABAs], short-acting beta-agonists [SABAs], steroids, COPD-related therapies), resource utilization, and variables from the first 30 days post discharge from the index hospitalization. A list of all variables used in the analyses is available in Additional file 1: Table S1.

#### Statistical analysis

Categorical variables were summarized using frequencies and percentages, and continuous measures using mean ± standard deviation (SD). Each predictor was run in a logistic regression model controlling for sex and age (with the exception that age was controlled for sex, and sex controlled for age). Since the nature of this study was exploratory, no adjustments for multiplicity were applied. Odds ratios (OR) and 95 % confidence intervals (CI) for the predictors, indicating their relative association with the likelihood of having an early or a late COPD-related rehospitalization post discharge from the index hospitalization were calculated. All data were analyzed using SAS^®^ Version 9.2 software (SAS Institute, Cary, NC, USA).

## Results

### Sample attrition and analysis group assignment

**Fig. 2:**
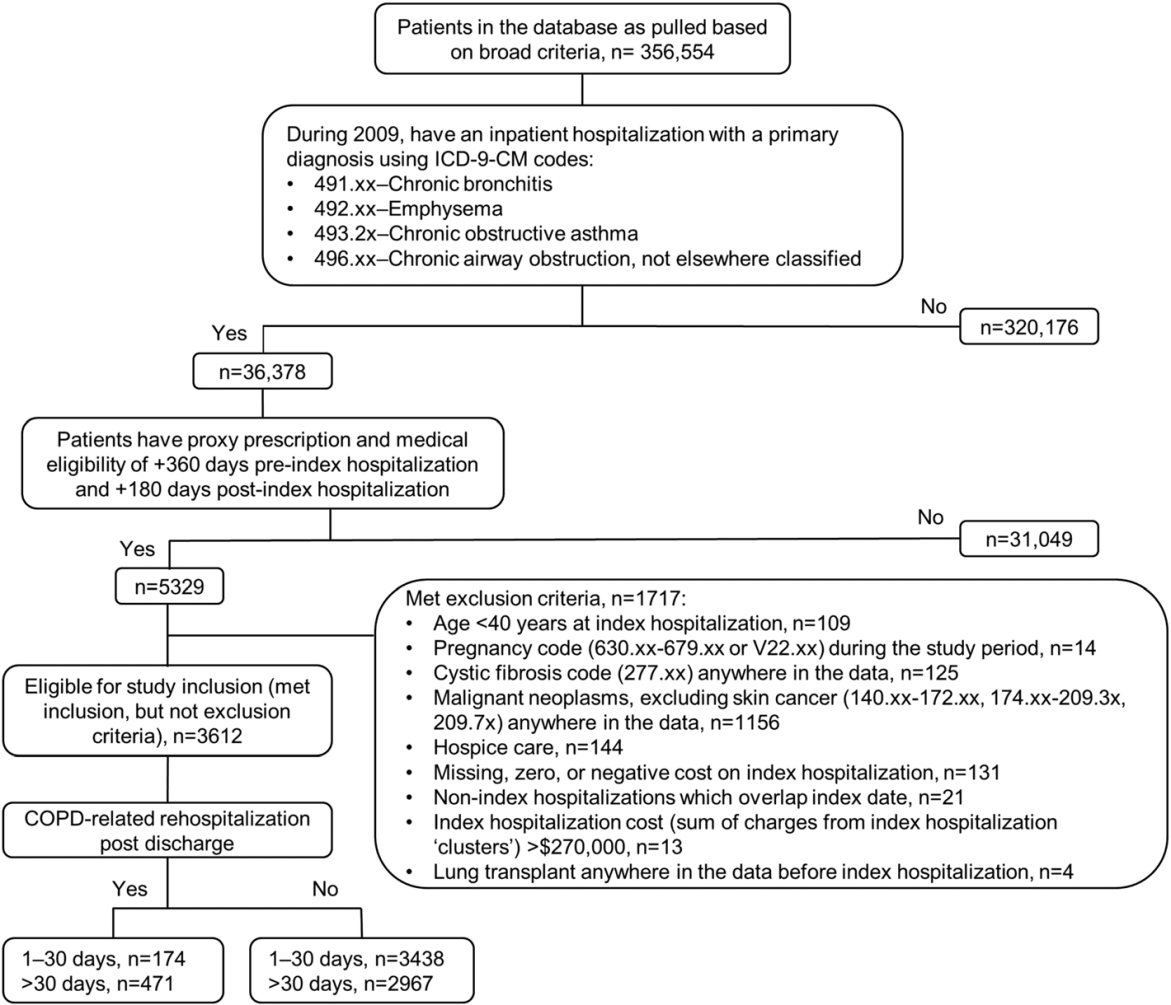
Sample attrition. ICD-9-CM, International Classification of Diseases, Ninth Revision, Clinical Modification

The sample attrition data are shown in Fig. 2. The initial data extraction process selected 356,554 patients with a diagnosis of interest in any field during 2008–2011. This number was reduced to 36,378 when only patients having a hospitalization with a primary diagnosis of interest during the 2009 enrollment window were selected. The proxy eligibility requirement excluded 31,049 patients and a further 1717 patients were omitted as a result of the exclusion criteria.

There were 3612 patients meeting all inclusion and no exclusion criteria. From post discharge to the end of the 180-day post-index period, 17.9 % (645/3612) of these patients had a COPD-related rehospitalization and 39.2 % (1417/3612) had an all-cause hospitalization. For the all-patient cohort, 4.8 % (174/3612) of patients had a first COPD-related rehospitalization within 30 days post discharge from the index hospitalization. Of the remaining 3438 patients (i.e., the late cohort), 13.7 % (471/3438) had a first COPD-related rehospitalization that occurred >30 days post discharge. In the all-patient cohort, patients with a first COPD-related rehospitalization during the first 30 days post discharge represented 36.2 % (174/481) of all patients experiencing at least one all-cause rehospitalization during this period. From >30 days post discharge to the end of the 180-day index period, the proportion of patients with a first COPD-related rehospitalization versus all-cause rehospitalization was 50.3 % (471/936 patients).

### Pre-index event variables

#### Demographic characteristics

Descriptions of the demographic characteristics for the all-patient and late cohorts are provided in Table 1. Independent logistic regression results indicated that in the all-patient cohort, younger age (OR = 0.98 for each year older [*P* = 0.007]) and Medicare insurance status (OR = 1.40 [*P* = 0.042]) were predictive of early COPD-related rehospitalization (i.e., occurring within 30 days post discharge from the index hospitalization). Whereas, male sex (OR = 1.34 [*P* = 0.005]), claims for smoking cessation products in the pre-index period (OR = 1.51 [*P* = 0.008]) and a pre-index indication of tobacco/smoking issues (i.e., diagnosis [history/use disorder], or HCPCS or CPT codes for past or present smoking or smoking cessation; OR = 1.74 [*P* < 0.001]) had greater odds of a COPD-related rehospitalization ≥30 days post discharge from the index hospitalization.

**Table 1:** Demographic characteristics of patients with COPD in the all-patient and late cohorts^a^

Characteristic	All-patient cohort (*n* = 3612)			Late cohort (*n* = 3438)		
	Early COPD-related rehospitalization			Late COPD-related rehospitalization		
	Yes (*n* = 174)	No (*n* = 3438)	*P*-value^b^	Yes (*n* = 471)	No (*n* = 2967)	*P*-value^b^
Age, years	64.2 ± 11.7	66.7 ± 12.1	0.007	66.3 ± 11.5	66.7 ± 12.2	0.513
Age group						
40–49 years	21 (12.1)	338 (9.8)	0.336	39 (8.3)	299 (10.1)	0.224
50–59 years	46 (26.4)	705 (20.5)	0.060	111 (23.6)	594 (20.0)	0.077
60–69 years	49 (28.2)	861 (25.0)	0.355	120 (25.5)	741 (25.0)	0.815
70–79 years	34 (19.5)	897 (26.1)	0.054	127 (27.0)	770 (26.0)	0.642
≥80 years	24 (13.8)	637 (18.5)	0.115	74 (15.7)	563 (19.0)	0.090
Male	64 (36.8)	1121 (32.6)	0.252	180 (38.2)	941 (31.7)	0.005
Medicare	97 (55.7)	1756 (51.1)	0.229	237 (50.3)	1519 (51.2)	0.723
Any indication of tobacco/smoking issues pre-index	70 (40.2)	1113 (32.4)	0.031	204 (43.3)	909 (30.6)	<0.001
Smoking cessation products pre-index	24 (13.8)	326 (9.5)	0.061	61 (13.0)	265 (8.9)	0.006
Region^c^						
North	30 (17.2)	511 (14.9)	0.391	81 (17.2)	430 (14.5)	0.125
South	73 (42.0)	1439 (41.9)	0.980	176 (37.4)	1263 (42.6)	0.034
East	39 (22.4)	743 (21.6)	0.802	113 (24.0)	630 (21.2)	0.177
West	27 (15.5)	594 (17.3)	0.548	81 (17.2)	513 (17.3)	0.961
Unknown region	5 (2.9)	151 (4.4)	0.336	20 (4.2)	131 (4.4)	0.868

Data are number of patients (%) or mean ± SD

^a^The all-patient cohort consisted of all patients in whom COPD-related rehospitalization was evaluated 1–30 days post discharge and the late cohort consisted of the subset of patients not rehospitalized early in whom late COPD-related rehospitalization was evaluated (>30 days post discharge to 180 days post-index hospitalization)

^b^ *P*-values are from chi-square tests for unadjusted percentages and independent *t* tests for unadjusted means

^c^Region is based on the region where the hospital is located

#### Comorbidities

**Fig. 3:**
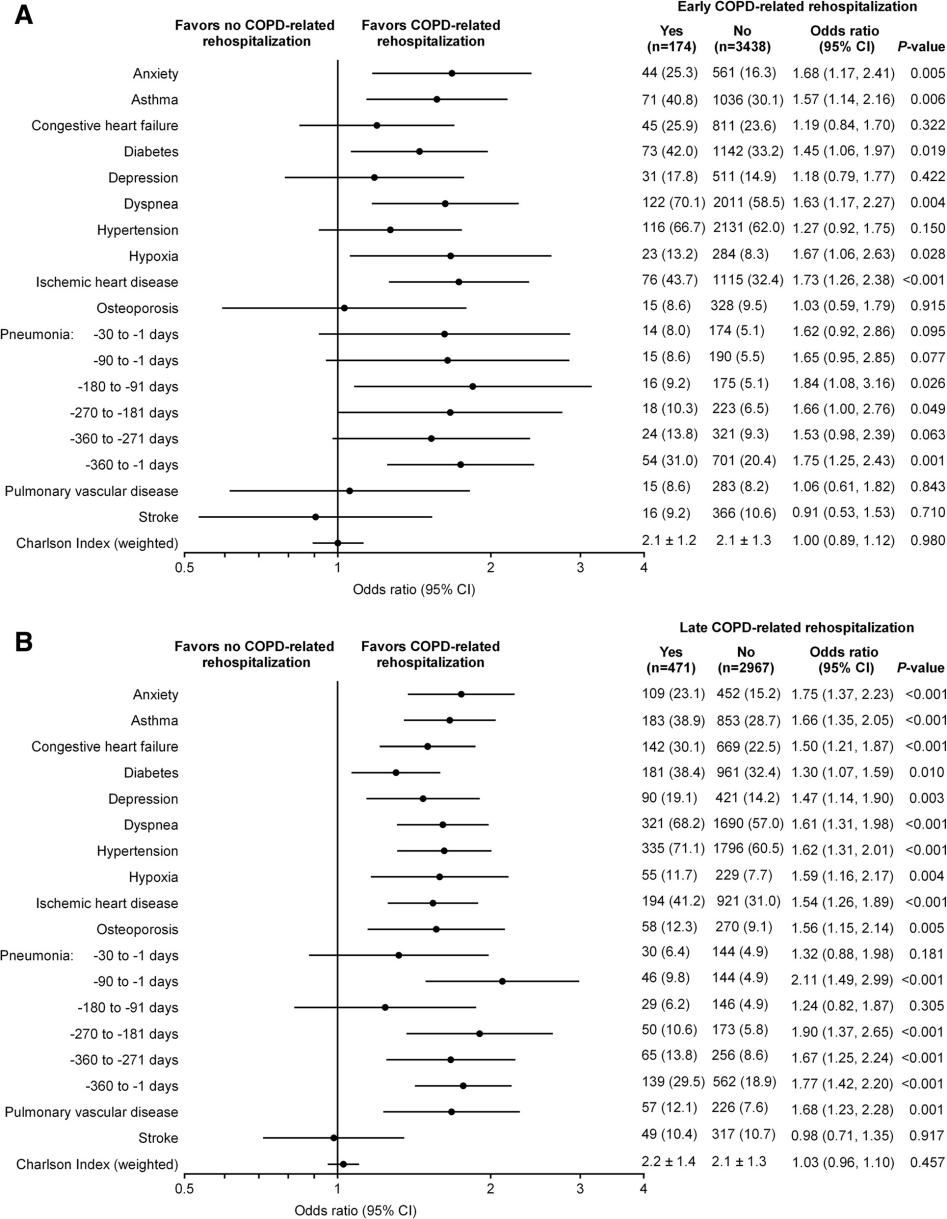
Diagnosis-based comorbidities identified in the 360-day pre-index period. Adjusted odds of (**a**) early and (**b**) late COPD-related rehospitalizations. The early cohort consisted of all patients in whom COPD-related rehospitalization was evaluated 1–30 days post discharge and the late cohort consisted of the subset of patients not rehospitalized early in whom late COPD-related rehospitalization was evaluated (>30 days post discharge to 180 days post-index hospitalization). Each factor was adjusted for sex and age in a logistic regression model. Data are number of patients (%) or mean ± SD. CI, confidence interval

Pre-index comorbidities, assigned based on diagnosis, and the adjusted ORs for COPD-related rehospitalizations are provided in Fig. 3. Anxiety, asthma, diabetes, dyspnea, hypoxia, ischemic heart disease, and a pneumonia code anytime during the 360-day pre-index period were predictors of early and late COPD-related rehospitalizations. In addition to these parameters, congestive heart failure, depression, hypertension, osteoporosis, and pulmonary vascular disease were also predictive of late COPD-related rehospitalization. The strongest predictor of late COPD-related rehospitalization was pneumonia within 90 days prior to the index hospitalization (OR = 2.11 [*P* < 0.001]). The OR estimates for early COPD-related rehospitalization were similar to those for late COPD-related rehospitalization, but fewer comorbidities achieved statistical significance. The Charlson comorbidity score was not significantly predictive of either an early or late COPD-related rehospitalization.

Data for treated comorbidities, assigned based on therapeutic categories of prescriptions dispensed during the 360-day pre-index period were explored. The categories that were predictive of an early COPD-related rehospitalization were greater use of blood products (i.e., blood derivatives and blood forming/coagulation agents; OR = 1.55 [*P* = 0.031]), cardiovascular drugs (i.e., blood pressure and cholesterol-lowering agents; OR = 1.41 [*P* = 0.038]) and hormones (i.e., steroids, estrogens, testosterones; OR = 1.55 [*P* = 0.008]). Presence of claims for antiarrhythmics was associated with a lower risk of a late rehospitalization (OR = 0.77 [*P* = 0.027]) and presence of claims for antiasthmatics (OR = 1.77 [*P* < 0.001]), gastrointestinal drugs (OR = 1.23 [*P* = 0.039]) and hormones (OR = 1.71 [*P* < 0.001]) were associated with a greater risk of late rehospitalization. A greater number of different drug classes per patient, including COPD classes (OR = 1.04 [*P* = 0.015]) was also predictive of a late COPD-related rehospitalization.

#### Respiratory-related therapies

**Fig. 4:**
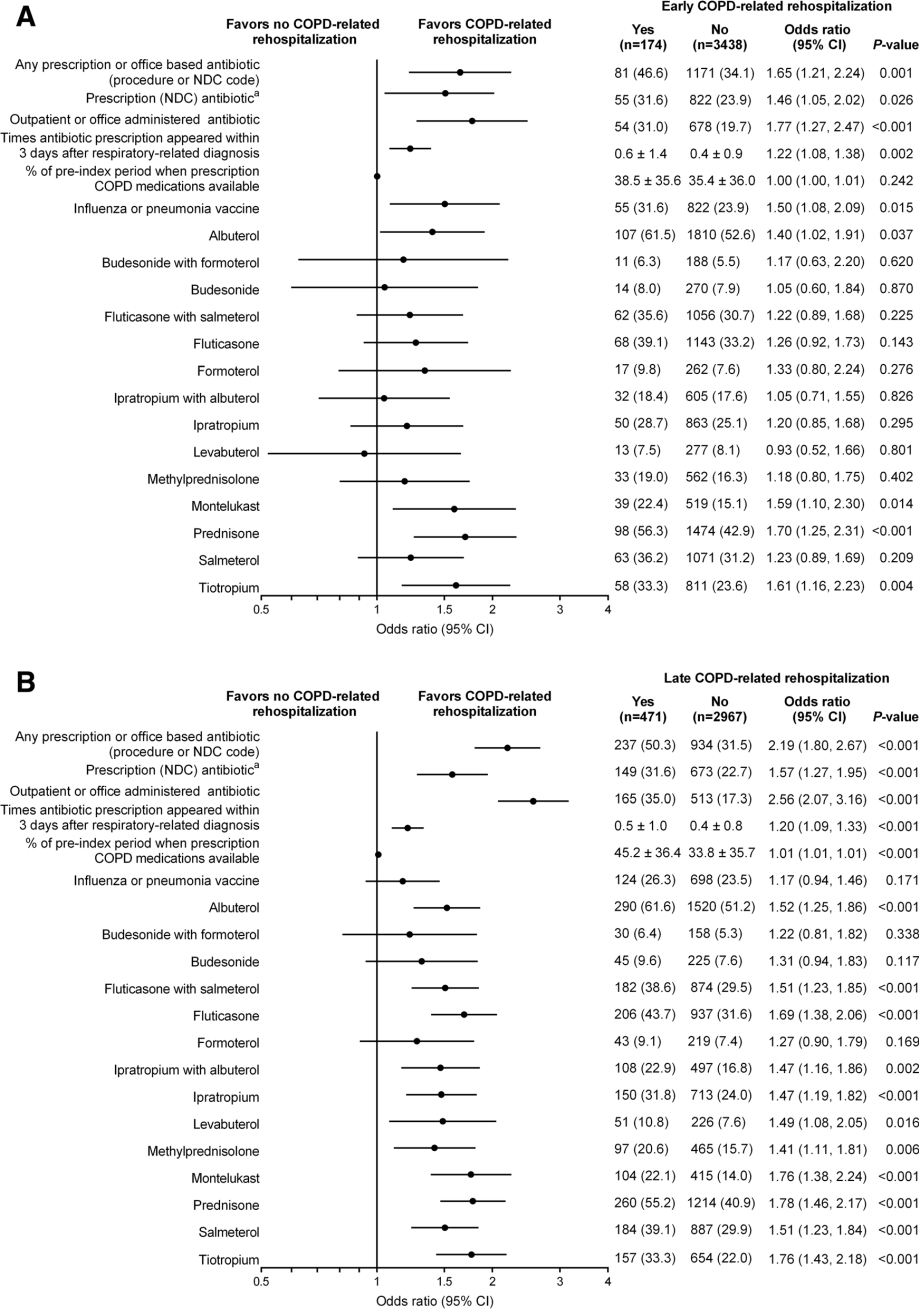
Outpatient prescription pharmacy utilization during the pre-index period. Adjusted odds of (**a**) early and (**b**) late COPD-related rehospitalizations. The early cohort consisted of all patients in whom COPD-related rehospitalization was evaluated 1–30 days post discharge and the late cohort consisted of the subset of patients not rehospitalized early in whom late COPD-related rehospitalization was evaluated (>30 days post discharge to 180 days post-index hospitalization). Each factor was adjusted for sex and age in a logistic regression model. Use is one or more fills unless otherwise indicated. The generic drugs include only non-injectable National Drug Code (NDC)-based drug claims appearing in the pharmacy database. All claims are collapsed by generic name, regardless of administration route. Only drugs used by ≥5 % of patients are reported. ^a^Antibiotics were only counted where they occur on or within 3 days following a respiratory-related diagnosis. Data are number of patients (%) or mean ± SD. CI, confidence interval

Pre-index outpatient respiratory-related prescription drug therapies (one or more fills) with adjusted ORs for COPD-related rehospitalization are provided in Fig. 4. For the drug variables, data are only presented for those generic entities for which ≥5 % of patients had claims.

Pre-index prescription (NDC billed) or office-based (HCPCS billed) antibiotics within 3 days of a COPD diagnosis were predictive of both early and late COPD-related rehospitalization (OR = 1.65 [*P* = 0.001] and OR = 2.19 [*P* < 0.001], respectively). The mean number of times that an antibiotic was prescribed within 3 days of a COPD diagnosis, suggestive of a moderate COPD exacerbation, was also predictive of an early and late COPD-related rehospitalization (OR = 1.22 [*P* = 0.002] and OR = 1.20 [*P* < 0.001], respectively).

Odds for an early COPD-related rehospitalization were higher for patients with pre-index availability of at least one SABA or LABA (OR = 1.60 [*P* = 0.021]) and non-inhaled steroids (OR = 1.68 [*P* = 0.001]). Pre-index inhaled steroid use was not predictive of early COPD-related rehospitalization. Patients had greater odds of a late COPD-related rehospitalization if they had claims in the pre-index period for at least one SABA or LABA (OR = 2.68), inhaled steroids (OR = 1.72), or non-inhaled steroids (OR = 1.75); all were statistically significant at *P* < 0.001. Data stratified by quarters prior to the index hospitalization were also examined, but did not reveal additional information.

Overall, presence of respiratory-related therapies was more predictive of a late than early COPD-related rehospitalization. The absolute differences for those with and without an early COPD-related rehospitalization were <10 % for all medications except prednisone, and were <10 % for all medications except albuterol, fluticasone, prednisone, and tiotropium in those with and without a late COPD-related rehospitalization. The adjusted ORs of COPD-related rehospitalization for oral prednisone use were 1.70 (*P* < 0.001) and 1.80 (*P* < 0.001) for early and late rehospitalizations, respectively; and were only slightly greater than those for inhaled medications.

A greater documentation of influenza vaccination was predictive of early COPD-related rehospitalization (OR = 1.50 [*P* = 0.015]), but not late COPD-related rehospitalization.

#### Healthcare utilization

**Fig. 5:**
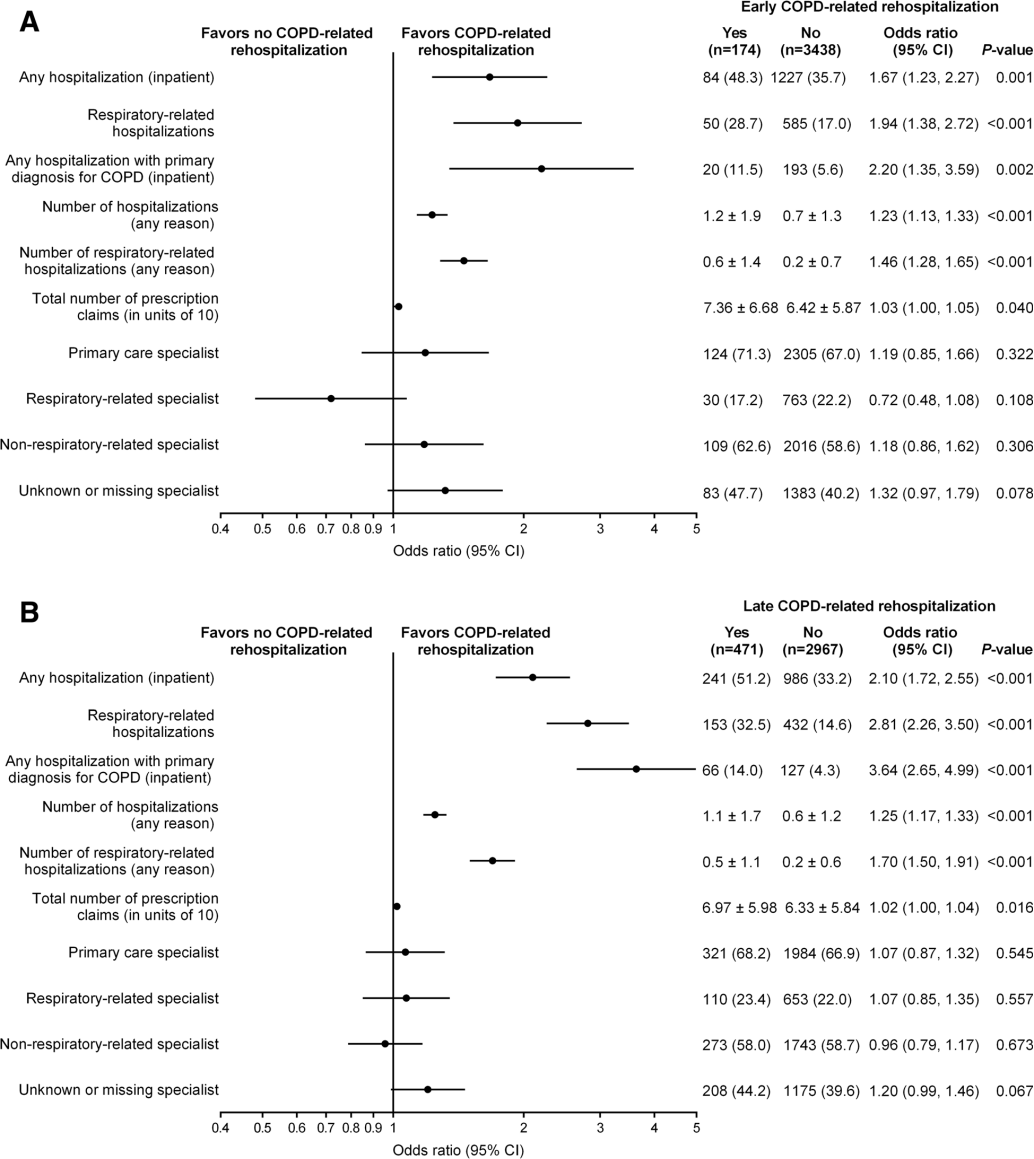
Pre-index period hospitalizations, outpatient clinic visits, and outpatient pharmacy claims. Adjusted odds of (**a**) early and (**b**) late COPD-related rehospitalization. The early cohort consisted of all patients in whom COPD-related rehospitalization was evaluated 1–30 days post discharge and the late cohort consisted of the subset of patients not rehospitalized early in whom late COPD-related rehospitalization was evaluated (>30 days post discharge to 180 days post-index hospitalization). Each factor was adjusted for sex and age in a logistic regression model. Data are number of patients (%) or mean ± SD. CI, confidence interval

Pre-index hospitalization resource use with adjusted ORs for COPD-related rehospitalization are provided in Fig. 5.

All-cause hospitalization in the pre-index period was strongly predictive of both early and late COPD-related rehospitalization (OR = 1.67 [*P* < 0.001] and OR = 2.10 [*P* < 0.001], respectively). A pre-index hospitalization with a primary diagnosis for COPD was shown to be an even stronger predictor of early and late COPD-related rehospitalization (OR = 2.20 [*P* = 0.002] and OR = 3.64 [*P* < 0.001], respectively).

Pre-index visits to primary care, respiratory-related and non-respiratory-related specialist were not predictive of either early or late COPD-related rehospitalization.

The total number of prescription claims (in units of 10) pre-index attained ORs that were statistically significant for early COPD-related rehospitalization (OR = 1.03 [*P* = 0.040]) and late COPD-related rehospitalization (OR = 1.02 [*P* = 0.016]).

### Index hospitalization

Treatment during the index hospitalization yielded few unexpected factors associated with COPD-related rehospitalization. Only the statistically significant findings are presented. Overall, more than 90 % of patients were treated with steroids and bronchodilators; it is likely that those not receiving these therapies were either misclassified, received drugs billed under bundled services, or were drug intolerant. Factors with higher odds of a late COPD-related rehospitalization included a higher number of COPD-related drug categories appearing in the index hospitalization (anticholinergics [tiotropium or ipratropium] and other bronchodilators by any route [see Additional file 1: Table S1 for generic drugs included] and steroids by inhalation: OR = 1.17 [*P* = 0.005]), and the use of steroids (any route: OR = 1.62 [*P* = 0.007], inhaled: OR = 1.42 [*P* < 0.001], and oral/injectable: OR = 1.46 [*P* = 0.015]) or nebulizers (OR = 1.65 [*P* = 0.007]). Treatment during the index hospitalization was not predictive of an early COPD-related rehospitalization.

Interestingly, index hospitalization at teaching hospitals (approximately one-third of the cohorts) was also associated with increased risk of late COPD-related rehospitalization (OR = 1.25 [*P* = 0.027]), but not early COPD-related rehospitalization.

### Post-index variables

**Fig. 6:**
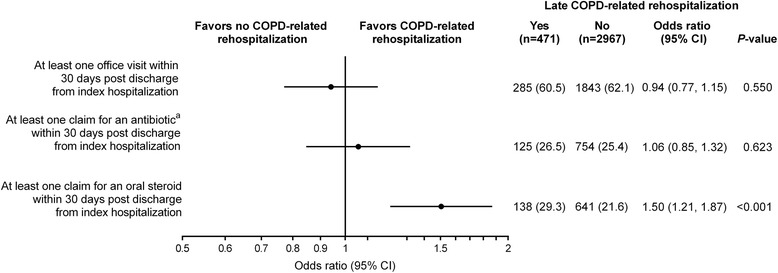
Office visits and medication claims in the 30 days post discharge from the index hospitalization. Adjusted odds of late COPD-related rehospitalization in the subset of patients not rehospitalized early in whom late COPD-related rehospitalization was evaluated (>30 days post discharge to 180 days post-index hospitalization). Each factor was adjusted for sex and age in a logistic regression model. ^a^Antibiotics were only counted where they occur on or within 3 days following a respiratory-related diagnosis. Data are number of patients (%). CI, confidence interval

Among the factors examined in the 30 days post-index hospitalization discharge, only one or more claims for an oral steroid was significantly associated with a late COPD-related rehospitalization (Fig. 6). Having an office visit in the first month after hospital discharge was not associated with a COPD-related rehospitalization >30 days post discharge (*P* = 0.550).

## Discussion

The aim of this exploratory analysis was to identify factors associated with COPD-related rehospitalization following a hospitalization for COPD. This retrospective analysis utilized a unique data source, which contained inpatient pharmacy data as well as medical and outpatient pharmacy claims data from the pre- and post-index hospitalization periods. Patients who experienced a COPD-related rehospitalization differed from those who did not experience a COPD-related rehospitalization on many pre-index hospitalization event variables including: a greater proportion of patients with some evidence of current or prior smoking; more frequent comorbidities (e.g., anxiety, asthma, diabetes, dyspnea, hypertension, hypoxia, ischemic heart disease, osteoporosis, and pneumonia); a greater use of COPD-related drug therapies (e.g., antibiotics, steroids, and bronchodilators); and more frequent hospitalizations (any reason, respiratory-related, or COPD-related). Most of these factors are likely to be markers of greater COPD severity. Although factors associated with late COPD-related rehospitalization were more likely to achieve statistical significance due to the greater length of time over which patients were observed (i.e., allowing for a greater number of patients with a rehospitalization), generally, among the factors that were available in the database, similar factors were found to be associated with early and late COPD-related rehospitalization.

These analyses identified several clinical parameters that might be useful for developing risk assessment systems for hospitalized COPD patients, and a few of the parameters associated with COPD-related rehospitalization are potentially modifiable. Pneumonia events and the use of antibiotics in the pre-index period were strongly associated with early and late COPD-related rehospitalization. A strong relationship between pneumonia and COPD rehospitalizations was also noted in an analysis of 2003–2004 Medicare claims data [16]. Regular daily use of azithromycin has been shown to reduce the frequency of COPD exacerbations, including those that result in hospitalization [19]. Perhaps, more attention to patients with COPD with a history of frequent bronchitis or pneumonia could reduce their COPD-related rehospitalization rates. Another potentially modifiable parameter is the generally poor adherence to COPD controller medications in the pre-index period. The observed association between respiratory medication use and an increased risk of COPD-related rehospitalization, most likely represents a bias by indication, wherein use of the medication is a marker for patients with more severe COPD. We found that adherence to COPD medications was poor even among those with early and late COPD-related rehospitalizations, with an average of only 38.5 and 45.2 %, respectively, of their pre-index period days covered by any respiratory medication. COPD controller medications have proven efficacy in reducing COPD exacerbations, and it is possible that better COPD treatment in this population could reduce both index hospitalizations and COPD-related rehospitalizations.

In our database, all-cause rehospitalization within 1–30 days post discharge and within 1 day post discharge to the end of the 180-day post-index period (13.3 % [481/3612 patients] and 39.2 % [1417/3612 patients], respectively) and COPD-related rehospitalization (4.8 % [174/3612 patients] and 17.9 % [645/3612 patients], respectively) were similar to, or lower than, those reported in other recent analyses. Our estimates may be lower, in part, since patients who died within 180 days of the index hospitalization were not included in our analysis. In an analysis of the 2003–2004 US Medicare population, the 30-day rehospitalization rates after a COPD admission were 22.6 % for all causes and 8.2 % primarily for COPD [16]. In a limited analysis of 2008 fee-for-service data, the 30-day rehospitalization rates after a COPD admission were 20.5 % for all causes and 7.1 % primarily for COPD [20]. In an analysis of another US insurance claims database of patients aged 40–65 years who were hospitalized for COPD between 2008 and 2010, the 30-day rehospitalization rates after a COPD admission were 8.2 % for all causes and 5.6 % primarily for COPD [21]. As in our study, this study also identified pre-index hospitalization healthcare utilization and comorbidity factors that were associated with an increased risk of all- cause and COPD-related rehospitalizations, even though their pre-index period was limited to 90 days [21]. In a sample of COPD admissions in the United Kingdom in 2008, the 90-day all-cause readmission rate was 34.2 % (2971/8677 patients) [22]. In a study of hospital readmissions for chronic medical conditions in Denmark and in the US Kaiser Permanente system from 2002 to 2007, all-cause 30-day readmission rates after a COPD hospitalization ranged from 20.7 to 24.1 % in Denmark, and from 19.4 to 21.4 % in Kaiser Permanente [23]. Another study of US claims data, using an analysis approach similar to ours, also found that factors associated with COPD severity at baseline (e.g., oxygen therapy, respiratory medicine use, and pulmonologist visits) were predictors of COPD-related readmission within 30 days [24].

In our analysis, having a prior hospitalization for COPD and the number of respiratory-related hospitalizations were strongly associated with greater odds of a rehospitalization. While we were not able to assess each patient’s total exacerbation experience, the finding does support the concept that there is a subset of patients who could be classified as “frequent exacerbators”; a classification that has been previously defined to include patients not only with severe airflow limitation, but also patients with moderate airflow limitation [25–27].

This study has some limitations. Administrative data were based on claim payments and not clinical treatment. We included as broad a range of variables as possible, based on what was available in the administrative database, but we recognize that there were many variables not captured by the database that might more accurately predict COPD-related rehospitalization. These include knowledge about the severity of each patient’s obstructive lung disease prior to and during the index hospitalization, assessment of partial pressure of carbon dioxide in arterial blood (PCO_2_), and the use of invasive and noninvasive ventilation. Additionally, inpatient stay information may not have completely captured hospitalization treatments; for example, patients may have had existing therapy on hand from prior to the index hospitalization. The extent to which coding within hospitals’ Charge Description Masters accurately reflect the care that a patient receives is unknown and it is possible that patients received care that was billed using terminology where it was not possible to associate the charge description with the correct category. Another limitation is that patients who died within 180 days of the index hospitalization were excluded from the analysis. This may create a bias that has an unknown effect. In this study we only focused on COPD-related rehospitalization and not on all-cause rehospitalization; some of the predictors of COPD-related rehospitalization may not be applicable to all-cause rehospitalization.

A large number of tests were performed and no correction was made for multiplicity. It is likely that some of the statistically significant tests were spurious. Since this analysis was exploratory, we elected to be more inclusive in order not to miss potential predictors. *P*-values and CIs are provided to allow readers to evaluate the level of significance. No multivariable models were assessed. No commentary is possible on the causal pathways or interrelationships among the variables.

A further limitation is that the analysis focused on a narrow definition of relapse, i.e., only COPD-related rehospitalizations were included; it is recognized that using rehospitalization as a marker for relapse underrepresents the total number of COPD relapses that occur, because patients not requiring rehospitalization were excluded. However, it does signify an important endpoint from an economic and humanistic perspective. It is also noteworthy that there is no definitive approach for identifying COPD-related hospitalizations. In the broadest sense, all hospitalizations may be affected by COPD.

## Conclusions

In this study, patients who experienced a COPD-related rehospitalization were significantly different from those who did not, on several pre-index hospitalization factors (smoking, comorbidities, COPD-related therapies, and more frequent hospitalizations, as well as more severe COPD). A strong predictor of a COPD-related rehospitalization was prior hospitalization, particularly with a primary COPD diagnosis. In-hospital and post-hospitalization variables had minimal effect on COPD-related rehospitalization risk as assessed in this study. Prospective analyses can focus on the parameters that were most strongly associated with COPD-related rehospitalization for the purposes of developing risk stratification systems, and for developing specific interventions that might reduce COPD-related rehospitalizations.

### Ethics approval and consent to participate

The retrospective SDI patient claims data were de-identified to ensure compliance with the Health Insurance Portability and Accountability Act (HIPAA) Privacy Rule standard for de-identification. Therefore, neither institutional review board approval nor informed patient consent were required for this study.

### Consent for publication

Not applicable.

### Availability of data and materials

Data for this analysis were from SDI Health, which was acquired by IMS Health (Plymouth Meeting, PA, USA) in 2011, and analyses were conducted under a data-use license agreement with IMS.

## Additional file

Additional file 1: Table S1. Analysis variables used for predicting COPD-related rehospitalization. (PDF 198 kb)

## Abbreviations

CI: confidence interval
COPD: chronic obstructive pulmonary disease
CPT: Current Procedural Terminology
HCPCS: Healthcare Common Procedure Coding System
ICD-9-CM: International Classification of Diseases, 9th Revision, Clinical Modification
LABA: long-acting beta-agonist
NDC: National Drug Code
OR: odds ratio
SABA: short-acting beta-agonist
SD: standard deviation
