# Supplementary material for: A retrospective analysis to identify predictors of COPD-related rehospitalization
Source: BMC Pulm Med. 2016 Apr 30;16:68. doi: 10.1186/s12890-016-0231-3 (PMC4851802; doi:10.1186/s12890-016-0231-3)
Supplement: Additional file 1: Table S1. — Analysis variables used for predicting COPD-related rehospitalization. (PDF 198 kb) [file 12890_2016_231_MOESM1_ESM.pdf]

Additional file 1: Table S1 Analysis variables used for predicting COPD-related rehospitalization

Category and timeframe	Description
Demographics: pre-index (–360 to –1 days)	Most frequent payer type of Medicare (vs all other [commercial insurance, Medicaid, cash, and missing/unknown])
	Smoking cessation products
	Any indication of tobacco/smoking issues (diagnosis [history/use disorder] or HCPCS or CPT codes for past or present smoking or smoking cessation)
Demographics: index hospitalization	Patient age at time of discharge from index event
	Gender
	Index hospital region – North (vs all other)
	Index hospital region – South (vs all other)
	Index hospital region – East (vs all other)
	Index hospital region – West (vs all other)
During index hospitalization	Anticholinergic (ipratropium or tiotropium) treatment (any route)
	Bronchodilator[a] treatment, excluding anticholinergics and inhaled steroids (any route)
	Markers for presence of secondary diagnoses for acute respiratory failure (518.81)[b] on the index hospitalization
	Presence of secondary diagnoses for heart failure (428.0x)[b]
	Number of days for index hospitalization
	Index admission through Emergency Room (vs all other)
	Number of beds in index event hospital: 1–99 (vs all other bed sizes)
	Number of beds in index event hospital: 100–199 (vs all other bed sizes)
	Number of beds in index event hospital: 200–299 (vs all other bed sizes)
	Number of beds in index event hospital: 300–499 (vs all other bed sizes)
	Number of beds in index event hospital: 500 and up (vs all other bed sizes)
	Number of beds in index event hospital: unknown (vs all other bed sizes)

	Number of COPD-related drug categories appearing in index hospitalization from the following: any route anticholinergics (ipratropium or tiotropium), any route other bronchodilators[a], inhaled steroids (range 0–3)
	Indications of nebulizer use during index hospitalization (Yes/No)
	Indications of oxygen therapy during index hospitalization (Yes/No)
	Steroid treatment (any route)
	Inhaled steroid treatment
	Non-inhaled (oral, injectable) steroid treatment
	Teaching (versus not)
	Sum of CDM resource charges for the index cluster
	Urban index hospital (vs rural)
Comorbidities[b] (by diagnosis): pre-index (–360 to –1 days)	Anxiety (300.0x, 300.2x, 300.3x, 306.9x, 308.xx, 309.2x, 309.4x, 309.9x)
	Asthma (493.xx)
	Charlson Comorbidity Index (weighted)
	Heart failure (428.xx)
	Diabetes (250.xx)
	Depression (296.2x, 296.3x, 296.9x, 309.0, 300.4, 311.xx)
	Infectious and parasitic diseases (001–139)
	Neoplasms (140–239)
	Endocrine (240–279)
	Blood diseases (280–289)
	Mental disorders (290–319)
	Nervous system and sense organs diseases (320–389)
	Circulatory system diseases (390–459)
	Respiratory system diseases (460–519)
	Digestive system diseases (520–579)
	Genitourinary system diseases (580–629)
	Skin and subcutaneous tissue diseases (680–709)
	Musculoskeletal system and connective tissue diseases (710–739)
	Symptoms, signs, and ill-defined conditions (780–799)
	Injury and poisoning (800–999)

	Supplementary classification (V01–V82)
	Dyspnea (786.xx)
	Hypertension
	Hypoxia (799.02)
	Ischemic heart disease
	Osteoporosis (733.0x)
	Pneumonia or influenza (480.xx–488.xx)
	Pulmonary vascular disease (415.xx–417.xx)
	Stroke (430.xx–438.xx)
Comorbidities[b] (by diagnosis): pre-index: –360 to –1 days, each quarter (Q1–Q4), and –30 to –1 days	Pneumonia or influenza (480.xx–488.xx)
Comorbidities[b] (by diagnosis): index hospitalization	Markers for presence of secondary diagnoses for pneumonia (480.xx–487.8x)
Comorbidities (by pharmaceutic treatment): pre-index (–360 to –1 days)	Number of days prescription COPD medications were available
	Antiarrhythmics (Yes/No)
	Antiasthmatics (Yes/No)
	Anticoagulants (Yes/No)
	Antihyperglycemics (Yes/No)
	Antineoplastics (Yes/No)
	Antiparkinson drugs (Yes/No)
	Autonomic drugs (Yes/No)
	Biologicals (Yes/No)
	Blood (Yes/No)
	Cardiac drugs (Yes/No)
	Cardiovascular drugs (Yes/No)
	Central nervous system drugs (Yes/No)
	Diuretics (Yes/No)
	Gastrointestinal drugs (Yes/No)
	Hormones (Yes/No)
	Immunosuppressants (Yes/No)
	Psychotherapeutics drugs (Yes/No)
	Thyroid preparations (Yes/No)

	Number of different drug classes per patient; including COPD-related classes
	Number of different drug classes per patient; excluding COPD-related classes
Prescription for LABA or SABA: pre-index (–360 to –1 days)	At least one SABA or LABA (any route)
	Number of quarters during the pre-index period with at least one claim for a LABA (i.e., dispensed, not based on daily markers)
	Number of quarters during the pre-index period with at least one claim for a SABA
Prescription for LABA or SABA: pre-index: –360 to –1 days; each quarter (Q1–Q4), and –30 to –1 days	At least one claim for a LABA, any route
	Percentage of days during each period where LABA were available (based on dispensing date and days' supply as recorded by the pharmacist)
	Ratio of percentage of days with LABAs to SABAs: formula = 100 × (number of LABA days/number of SABA days). Where there was no use of one or the other a zero was assigned
	Indicator variables (Yes/No) for each period where there were any SABA claims
	Percentage of days during each period where SABA were available (based on dispensing date and days' supply as recorded by the pharmacist)
Prescription for steroids: pre-index (–360 to –1 days)	Number of quarters where there was at least one claim for a steroid inhaler
	Number of quarters where there was at least one claim for a non-inhaled steroid
	Steroid (any route)
Prescription for steroids: pre-index: –360 to –1 days, each quarter (Q1–Q4), and -30 to –1 days	Indicator variables (Yes/No) for presence of any steroid inhaler claims
	Percentage of days during each period where steroid inhalers were available (based on dispensing date and days' supply as recorded by the pharmacist)
	Indicator variables (Yes/No) for presence of any non-inhaled steroid claims
	Variables for the percentage of days during each period where non-inhaled steroid were available (based on dispensing date and days' supply as recorded by the pharmacist)
	All COPD-related study medicines

COPD-related drug availability: pre-index: –360 to –1 days, Q12, and Q34	Albuterol
	Arformoterol
	Beclomethasone
	Budesonide with formoterol
	Budesonide
	Ciclesonide
	Cortisone acetate
	Cromolyn
	Dexamethasone
	Dyphylline
	Flunisolide
	Fluticasone with salmeterol
	Fluticasone
	Formoterol
	Hydrocortisone
	Ipratropium with albuterol
	Ipratropium
	Levalbuterol
	Metaproterenol
	Methylprednisolone
	Mometasone
	Montelukast
	Pirbuterol
	Prednisolone
	Prednisone
	Salmeterol
	Terbutaline
	Tiotropium
	Triamcinolone
	Zafirlukast
	Zileuton
Other respiratory treatments of interest: pre-index (–360 to –1 days)	At least one prescription or office-based antibiotic (procedure or NDC code)
	Prescription (NDC) antibiotic
	Outpatient or office-administered antibiotic

	Number of times a prescription for antibiotics appeared within 3 days after a respiratory-related diagnosis
	Percent of the pre-index period during which prescription COPD medications were available
	Influenza or pneumonia vaccine
Costs utilization: pre-index (–360 to –1 days)	At least one hospitalization (any reason)
	Hospitalization with a COPD ICD-9-CM code present in the primary diagnosis field (Yes/No)
	Hospitalization with a respiratory-related ICD-9-CM code in the primary diagnosis field (Yes/No)
	Number of hospitalizations (any reason)
	Number of respiratory-related hospitalizations
	Primary care specialist during (Yes/No)
	Respiratory-related specialist (Yes/No)
	Total number of prescription claims in units of 10 (i.e., per 10 prescriptions)
	Unknown or missing specialist (Yes/No)
	Non-respiratory-related specialist (Yes/No)
1–30 days post-discharge from index hospitalization	Presence of at least one claim for an antibiotic (Yes/No)
	Presence of at least one office visit (Yes/No)
	At least one claim for an oral steroid (Yes/No)

CDM, Charge Description Master; CPT, Current Procedural Terminology; HCPCS, Healthcare Common Procedure Coding System; LABA, long-acting beta-agonist, SABA, short-acting beta-agonist, NDC, National Drug Code; Q1, –360 to –271 days pre-index date; Q2, –270 to –181 days pre-index date; Q3, –180 to –91 days pre-index date; Q4, –90 to –1 days pre-index date; Q12, –360 to –181 days pre-index date; Q34, –180 to –1 days pre-index date.

[a]Albuterol, aminophylline, arformoterol, dyphylline, ephedrine, formoterol, levalbuterol, metaproterenol, montelukast, salmeterol, terbutaline, theophylline, zafirlkulast, zileuton.

[b]Disease classifications are based on International Classification of Diseases, 9th Revision, Clinical Modification (ICD-9-CM) codes.
